# Screening for early Alzheimer’s disease: enhancing diagnosis with linguistic features and biomarkers

**DOI:** 10.3389/fnagi.2024.1451326

**Published:** 2024-09-23

**Authors:** Chia-Ju Chou, Chih-Ting Chang, Ya-Ning Chang, Chia-Ying Lee, Yi-Fang Chuang, Yen-Ling Chiu, Wan-Lin Liang, Yu-Ming Fan, Yi-Chien Liu

**Affiliations:** ^1^Department of Neurology, Cardinal Tien Hospital, Taipei, Taiwan; ^2^Department of Speech-Language Pathology and Audiology, National Taipei University of Nursing and Health Sciences, Taipei, Taiwan; ^3^Miin Wu School of Computing, National Cheng Kung University, Tainan, Taiwan; ^4^Academia Sinica, Taipei, Taiwan; ^5^Institute of Public Health, College of Medicine, National Yang Ming Chiao Tung University, Taipei, Taiwan; ^6^International Health Program, College of Medicine, National Yang Ming Chiao Tung University, Taipei, Taiwan; ^7^Health Innovation Center, National Yang Ming Chiao Tung University, Taipei, Taiwan; ^8^Department of Medical Research, Far Eastern Memorial Hospital, Taipei, Taiwan; ^9^Graduate Program in Biomedical Informatics and Graduate Institute of Medicine, Yuan Ze University, Taoyuan, Taiwan; ^10^Graduate Institute of Clinical Medicine, National Taiwan University, Taipei, Taiwan; ^11^School of Medicine, Fu Jen Catholic University, Taipei, Taiwan; ^12^Department of Nuclear Medicine, Cardinal Tien Hospital, Taipei, Taiwan

**Keywords:** Alzheimer’s disease, linguistic features, cognitive impairment, amyloid-*β*, hippocampal volume, speech analysis

## Abstract

**Introduction:**

Research has shown that speech analysis demonstrates sensitivity in detecting early Alzheimer’s disease (AD), but the relation between linguistic features and cognitive tests or biomarkers remains unclear. This study aimed to investigate how linguistic features help identify cognitive impairments in patients in the early stages of AD.

**Method:**

This study analyzed connected speech from 80 participants and categorized the participants into early-AD and normal control (NC) groups. The participants underwent amyloid-*β* positron emission tomography scans, brain magnetic resonance imaging, and comprehensive neuropsychological testing. Participants’ speech data from a picture description task were examined. A total of 15 linguistic features were analyzed to classify groups and predict cognitive performance.

**Results:**

We found notable linguistic differences between the early-AD and NC groups in lexical diversity, syntactic complexity, and language disfluency. Using machine learning classifiers (SVM, KNN, and RF), we achieved up to 88% accuracy in distinguishing early-AD patients from normal controls, with mean length of utterance (MLU) and long pauses ratio (LPR) serving as core linguistic indicators. Moreover, the integration of linguistic indicators with biomarkers significantly improved predictive accuracy for AD. Regression analysis also highlighted crucial linguistic features, such as MLU, LPR, Type-to-Token ratio (TTR), and passive construction ratio (PCR), which were sensitive to changes in cognitive function.

**Conclusion:**

Findings support the efficacy of linguistic analysis as a screening tool for the early detection of AD and the assessment of subtle cognitive decline. Integrating linguistic features with biomarkers significantly improved diagnostic accuracy.

## 1 Introduction

Alzheimer’s disease (AD) is a primary neurodegenerative disorder predominantly affecting individuals over 60 years old. Studies have found that approximately 40% of AD risk factors can be modified in the early stages of the disease (Livingston et al., 2020), presenting an opportunity for interventions aiming to prevent or delay cognitive decline. Neuropsychological tests have traditionally focused on assessing episodic memory recall and general cognitive functions, which have demonstrated acceptable accuracy in AD detection (Arevalo-Rodriguez et al., 2021). Additionally, advancements in AD biomarkers such as hippocampus volume (Chupin et al., 2009; Liu et al., 2020) and amyloid burden (Morris et al., 2016) have substantially enhanced accuracy up to 90% in identifying early pathological changes. Clinical evidence has suggested that positive biomarkers increase but do not predict the likelihood of progression (Dubois et al., 2021). In research involving a cohort of cognitively unimpaired individuals, subjects with elevated levels of brain amyloid exhibited no signs of cognitive decline until approximately 3–4 years from the baseline assessment (Donohue et al., 2017). This result highlights the complexity of predicting AD progression, which requires a multifaceted approach that includes the assessment of biomarkers and the identification of subtle cognitive impairments.

AD weakens episodic memory, which worsens as AD progresses. The deterioration of other cognitive functions, including language, is also observed. In analyses of connected speech in neurodegenerative diseases (Boschi et al., 2017; Kavé and Goral, 2016; López-de-Ipiña et al., 2013), AD has been characterized by lexical-semantic deficits, such as difficulties in word retrieval and reduced lexical diversity. These distinctions highlight the potential for a tailored diagnosis based on specific language profiles associated with AD. Researchers have used linguistic and acoustic features of speech to distinguish patients with early-AD from healthy older adults, reporting high accuracy rates for AD diagnosis ranging from 80 to 97% (Fristed et al., 2022; Lindsay et al., 2021; Martínez-Nicolás et al., 2021). The speech analysis approach not only facilitates the early detection of cognitive decline but also serves as an alternative that is more accessible and noninvasive than biomarkers.

In connected speech, changes in language usage and speech patterns can be observed well before the clinical diagnosis of AD (Karr et al., 2018; Mueller et al., 2018a). Studies have shown that language integrity gradually declines in the early stages of AD (Ahmed et al., 2013b; Beltrami et al., 2018), significantly affecting lexical-semantic and syntactic complexity in individuals with mild cognitive impairment (MCI). Studies have also achieved high classification accuracy by using a large set of linguistic features, but such accuracy can be maintained by selecting only the most essential features (Balagopalan et al., 2021; Clarke et al., 2021; Ostrand and Gunstad, 2021). Hence, this research aims to explore linguistic anomalies as early signals of cognitive impairments to facilitate early AD diagnosis. Our strategy involves a focused approach to identifying critical linguistic features from existing research to ensure accurate detection without the need for an extensive feature set.

This study used picture description task to elicit linguistic features of connected speech. This task is commonly used to evaluate connected speech (Clarke et al., 2021; Filiou et al., 2020; Hernández-Domínguez et al., 2018) and requires participants to describe what they see in images without the effort of memorizing previously heard words or narratives. Filiou et al. (2020) thoroughly examined the effectiveness of this task in distinguishing language production subtleties, focusing on various linguistic aspects such as fluency, semantics, syntax, and lexicon to differentiate the phases of AD and MCI. In individuals with AD, the study revealed significant semantic and lexical deficits characterized by a reduction in the use of precise, content-rich vocabulary and a shift toward generic words such as pronouns (Kavé and Goral, 2016). Additionally, a significant decline was observed in semantic units associated with actions and subjects, indicating a reduction in the use of verbs and syntactic complexity, which are critical indicators of AD (Ahmed et al., 2013a). This pattern has been observed in patients with MCI, for example, often replacing specific nouns with pronouns (Mueller et al., 2016; Ostrand and Gunstad, 2021) and using indefinite articles and empty expressions (Ahmed et al., 2013b; Mueller et al., 2018a). These linguistic patterns suggest a decline in semantic integrity, indicating cognitive decline in its early stages.

During speech analysis, syntactic complexity has often been used as a marker for cognitive decline. Cognitive challenges such as difficulties in word retrieval, executive function problems, and working memory constraints can be directly linked to the inability to form complex utterances and meaningful sentences (Fraser et al., 2015). In such a context, mean length of utterance (MLU) is a critical metric, providing insights into the complexity of an individual’s language. In MLU, longer utterances typically represent higher complexity and are commonly used to assess language development and identify potential disorders (Ash et al., 2011; Sadri Mirdamadi and De Jong, 2015). In the MCI field, a notable shift is observed: affected individuals’ number of grammatically accurate sentences decreases, often replaced with simple verb tenses (Ahmed et al., 2013b). This change in verb usage is a crucial indicator for clinicians to classify the syndromic trajectory of language decline, especially in the early stages of AD. Mueller et al. (2018b) found that patients with MCI tend to use pronouns and verbs frequently in their speech, indicating a shift toward the construction of noun phrases without nouns. Additionally, individuals with mild AD often show a reduction in verb usage and an increase in filled pauses, considered hallmark indicators of language degradation (Ahmed et al., 2013a; Sajjadi et al., 2012; Yuan et al., 2021).

Research has revealed that individuals with AD pause for long periods silently and more frequently (Gayraud et al., 2011; Hoffmann et al., 2010; Pistono et al., 2019) in their speech. Although brief pauses of less than 1 s are a natural part of spoken language, those beyond 2 s can significantly interrupt the conversational flow, suggesting cognitive impairment or decline (Nasreen et al., 2021; Wang et al., 2022). Additionally, patients with MCI exhibit more pauses at a greater rate than healthy controls (Roark et al., 2011; Toth et al., 2018). Although the average duration of these pauses is generally longer in affected individuals than in nonaffected individuals (Pistono et al., 2016), some studies have reported no significant differences in pause duration between these groups (Roark et al., 2011; Sluis et al., 2020). Research has linked silent pauses to episodic memory retrieval, indicating that individuals rely on these breaks for memory retrieval and planning. Pistono et al. (2019) observed more frequent pauses among patients in the prodromal to mild stages of AD who scored higher on semantic fluency tests. Pauses May serve as a compensatory mechanism for navigating lexical-semantic and memory challenges during the early stages of AD and MCI. These insights have substantially strengthened the understanding of how AD and MCI affect speech fluency and have potential to evaluate cognitive decline through speech pattern analysis.

This study examined narrative speech data from participants performing the picture description task. The aim was to identify linguistic features of cognitive impairment in individuals with MCI and mild AD that differentiate them from normal controls (NCs). Several key strategies were implemented to ensure applicable results. Clinical diagnoses were confirmed using biomarkers. The control group comprised individuals with negative amyloid-*β* PET scans and normal neuropsychological test scores, providing a clear contrast with the cognitively impaired group. Participants were monitored at a memory clinic for at least 2 years, allowing for an observation of symptom progression and more accurate diagnoses. This study was unique because it focused on a Chinese-speaking cohort, addressing a gap in the literature predominantly based on English-speaking populations. To mitigate cultural bias often observed in the traditional “cookie theft” task, culturally relevant image stimuli were employed to elicit speech samples. This approach aimed to encourage more natural engagement from participants, potentially yielding richer and more representative language samples. By addressing cultural and linguistic specificities, this study aimed to contribute valuable insights to the field of early AD detection in diverse populations.

## 2 Materials and methods

### 2.1 Participants

Picture descriptions were produced by 80 participants from the memory clinic of Cardinal Tien Hospital in Taipei, Taiwan, between September 2019 and August 2023. Inclusion criteria for all participants were age 60–90 years and at least 6 years of education. The early-AD group had no history of neurological or psychiatric disorders. The control group comprised hospital volunteers. All participants demonstrated the absence of objective cognitive deficits on neuropsychological testing and maintained biannual follow-up appointments at the memory clinic for 24 months. A neurologist (YCL) evaluated these participants according to the research frameworks of the National Institute on Aging–Alzheimer’s Association diagnostic criteria. The participants were classified into early-AD and NC groups based on their Clinical Dementia Rating (CDR) scores, brain magnetic resonance imaging (MRI), and amyloid-*β* PET scans conducted within 2 years before recruitment. Brain MRI was used to exclude other neurological conditions such as brain tumors, and amyloid-β PET scans were primarily evaluated using visual rating to confirm amyloid positivity.

The early-AD group comprised 48 individuals: 34 with aMCI and 14 with mild AD. A CDR score of 0.5 indicates MCI, and a score of 1 indicates mild AD. The CDR Sum of Boxes (CDR-SB) was used to monitor the patient’s disease severity progression. An increase of 1 or more in CDR-SB scores over 1 year generally indicates clinical progression (i.e., a transition from MCI to more advanced stages of cognitive decline). Their MMSE (mini-mental state examination) scores were between 20 to 26. The control group comprised 32 individuals who scored 0 on the CDR and greater than 26 on the MMSE, and Table 1 shows the detailed demographic information of these participants.

**Table 1:** Participants’ demographic characteristics and clinical features.

	NC, *N* = 32	Early-AD, *N* = 48	*p*-value
Age, year	72.23 (6.03)	75.02 (6.58)	**0.046**
Education, year	13.66 (3.49)	10.03 (4.86)	**<0.001**
Gender, no. female	14 / 32 (44%)	31 / 48 (65%)	0.11
MMSE	28.94 (1.27)	21.19 (5.30)	**<0.001**
CDR-SB	0.02 (0.09)	3.20 (2.49)	**<0.001**
Change in CDR-SB score over 2 years	0.09 (0.39)	1.46 (1.85)	**<0.001**
Progressive	0 / 32 (0%)	36 / 48 (75%)	**<0.001**
*Biomarkers*			
Amyloid positivity, no. positive (%)	0 / 32 (0%)	48 / 48 (100%)	**<0.001**
Standardized uptake value	1.08 (0.08)	1.47 (0.19)	**<0.001**
Hippocampus, volume, cm^3^	5.12 (0.48)	4.36 (0.72)	**<0.001**
ApoEε4 carrier, no.(%)	4 / 30 (13%)	26 / 45 (58%)	**<0.001**
*Cognitive functions, mean (SD)*			
*Attention/information processing speed (standard score)*			
DS	12.06 (2.91)	7.03 (4.18)	**<0.001**
*Episodic memory (standard score)*			
WLM-I	13.59 (2.42)	5.63 (3.48)	**<0.001**
WLM-II	13.56 (2.59)	4.82 (3.52)	**<0.001**
*Executive function (raw score)*			
CTT-1, sec	59.13 (19.45)	159.82 (103.19)	**<0.001**
CTT-2, sec	123.89 (37.04)	262.03 (131.21)	**<0.001**
VF	17.19 (4.42)	9.88 (4.40)	**<0.001**
*Language (raw score)*			
BNT	24.88 (2.73)	19.31 (4.71)	**<0.001**

Values are presented as means (SDs). MMSE, mini-mental status examination; DS, summary score of digit span; WLM-1, Logical Memory subtest of Wechsler Memory Scale (part 1, immediate recall); WLM-2, Logical Memory subtest of Wechsler Memory Scale (part 2, delayed recall); CTT-1, Color Trails Test part 1 (with ascending number sequence only), CTT-2, Color Trails Test part 2 (with alternating color change); VF, animal verbal fluency score; BNT, Taiwanese version of Boston Naming Test (30 item). Bold values indicate statistically significant differences between the NC and Early-AD groups.

### 2.2 Neuropsychological testing

The participants underwent a battery of neuropsychological tests to assess specific domains of their cognitive function. The Digit Span (DS) subtests and Digit Symbol Substitution (DSS) subtests of the Wechsler Adult Intelligence Scale-IV evaluated auditory attention and working memory. Memory was evaluated using the Logical Memory subtests from the Wechsler Memory Scale-III, which include immediate recall (WLM-I) and delayed recall (WLM-2). Color Trails Tests 1 and 2 (CTT-1, CTT-2), the Stroop Color and Word Test, and the animal category fluency task (i.e., VF) were employed for executive function. The Taiwanese version of the Boston Naming Test (BNT; 30-item version) was used for the language domain.

### 2.3 Acquisition and processing of brain MRI

All participants underwent whole-brain MRI scans (3.0 T, MAGNETOM Skyra, Siemens, Taipei, Taiwan). The MRI acquisition protocol was as follows: whole-brain axial and sagittal T2-weighted fluid-attenuated inversion recovery (FLAIR) sequence (FLAIR repetition time [TR]/time to echo [TE] = 3550/98 ms) with a slice thickness of 5 mm, axial T1-weighted sequence (TR/TE = 2200/3 ms) with a slice thickness of 1 mm, and high-resolution coronal T1-weighted three-dimensional (3D) magnetization-prepared rapid gradient-echo image (TR/TE = 2200/5 ms) with a slice thickness of 1 mm.

### 2.4 Evaluation of hippocampal volume

Hippocampal volume was estimated using high-resolution coronal T1-weighted 3D images. We employed the PNEURO module of PMOD software (version 4.1, PMOD Technologies, Zurich, Switzerland) and implemented the following steps for our analysis: First, we denoised the uploaded T1-weighted MRI scans; second, we performed segmentation to identify gray matter, white matter, and cerebrospinal fluid and split the hemispheres; third, we parcellated the brain structures and presented the brain contours; and fourth, we calculated the hippocampal volume of interest.

### 2.5 Amyloid-*β* PET assessment

Brain amyloid PET was used in this study to determine the amyloid burden in the participants. Florbetaben was used as the amyloid PET tracer. The positivity of amyloid PET was based on a consensus meeting (with one neurologist and three nuclear medicine specialists). Quantitative analysis of PET images was performed using quantitative analysis via PMOD (version 4.1, PMOD Technologies, Zurich, Switzerland). In detail, T1-weighted MRI scans and the PET scans delineated the regions of interest on the MRI scans in accordance with the Hammers template and superimposed them onto the dynamic PET scans to obtain regional time activity curves [27]. Next, we obtained nondisplaceable binding potential images by using cerebellum gray matter as a reference region. Finally, we calculated the volume-weighted mean cortical amyloid-β load by using the Hammers brain atlas, which comprises all cortical regions (i.e., frontal, temporal, parietal, occipital, and cingulate cortices). We collected late-phase images 50 min after the tracer injection and then performed a second scan with a duration of 20 min to measure amyloid-β burden. We also determined the standardized uptake value ratio (SUVR) for the entire brain and in specific brain regions (i.e., frontal, parietal, temporal, occipital, cingulate, and insular). A cutoff value of 1.19 (specificity 91.83%; sensitivity 94.54%) was established using receiver operating curve (ROC) analysis, with visual read results as the standard reference.

### 2.6 Recording and extraction of linguistic features

**Figure 1:**
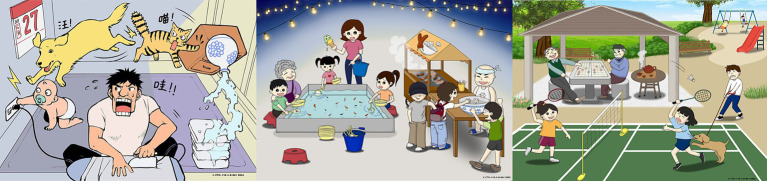
A set of three pictures on Taiwanese culture as visual stimuli to elicit descriptive responses from study participants for speech analysis.

This study employed a picture description task to elicit connected speech and used a digital recorder to record the responses. Participants described a set of three images depicting Taiwanese culture (Figure 1), with the instruction to report everything they observed in each image. The evaluators refrained from providing feedback but encouraged participants to elaborate if their responses were insufficient. Three trained graduate students transcribed these recordings, which were reviewed and refined by a language therapist on our team. The transcribers were unaware of the clinical conditions, neuropsychological testing results, or neuroimaging biomarkers and only recorded words spoken by the participants and the total number of words produced. The remaining words were segmented into utterances and annotated as pauses, filled pauses (e.g., “uh,” “um,” “er,” and “ah”), repetitions, and revisions. Utterances were manually segmented based on semantic and syntactic completeness and pause duration. A minimum pause duration of 120 ms was used as a guideline for potential utterance boundaries. However, the final determination of utterance boundaries also considered semantic coherence and syntactic completeness. Filled pauses were not considered words, and instances of immediate repetition or perseveration of the same word or utterance were excluded (e.g., “They brew-brew a pot of tea” was recorded as “They brew a pot of tea”). Words were grouped by part of speech and tagged using the Chinese Knowledge and Information Processing Lab.^1^ A total of 15 linguistic features spanning three speech aspects—lexical content, syntactic complexity, and speech disfluency (Table 2)—were extracted from each image, and their averages were calculated for statistical analysis.

**Table 2:** Definitions of linguistic measures and analysis of connected speech production.

Type	Linguistic features	Definition	NC	Early-AD	*p*-value
Lexical content	Total Words (TW)	Total word count	113.74 (47.40)	112.08 (63.07)	0.70
	Unique Words (UW)	Number of unique words	60.08 (18.52)	51.86 (19.96)	0.30
	Type-to-Token Ratio (TTR)	UW divided by TW	0.56 (0.08)	0.51 (0.11)	0.11
	Content Words (CW)	Number of content words	53.03 (18.84)	49.32 (25.45)	0.90
	Content Density (CD)	CW divided by TW	0.48 (0.05)	0.46 (0.06)	0.10
	Content Word Frequency (CWF)	Mean word frequency of all content words	857.50 (184.46)	1,024.84 (264.60)	0.07
Syntactic complexity	Utterance (U)	Number of utterances	21.68 (9.85)	29.81 (21.42)	0.00**
	Sentence (S)	Number of sentences	13.10 (4.83)	11.92 (6.37)	0.60
	Mean Length of Utterance (MLU)	Mean characters per utterance	8.11 (3.02)	5.53 (1.62)	<0.001***
	Mean Length of Sentence (MLS)	Mean words per sentence	9.34 (1.33)	11.16 (3.45)	0.01*
	Passive Construction Ratio (PCR)	Number of passive constructions divided by U	0.03 (0.03)	0.01 (0.02)	<0.001***
	Verb Ratio (VR)	Number of verbs divided by CW	0.22 (0.03)	0.22 (0.03)	0.40
	Pronoun Ratio (PR)	Number of pronouns divided by CW	0.03 (0.02)	0.05 (0.02)	0.04*
Disfluency	Filler Ratio (FR)	The number of filled pauses uttered divided by the total verbal output (the sum of TW and the number of filled pauses)	0.05 (0.02)	0.06 (0.05)	0.15
	Long Pauses Ratio (LPR)	Number of long pauses divided by the total verbal output	0.00 (0.01)	0.02 (0.02)	0.01*

Values are presented as means (SDs). Asterisks denote significant impairment relative to normal controls (NCs) at *p < 0.05, **p < 0.01, ***p < 0.001. The Linguistic feature column contains the name of the feature. The definition column explains how the feature is calculated. *p*-values from linear regression models adjusted for age and education. Bold values indicate statistically significant differences between the NC and Early-AD groups.

Various methods were adopted to evaluate lexical content. The *total number of words (TW)* and *unique words (UW)* produced from three images were determined. Lexical richness was assessed using *type-token ratio (TTR)*. Nouns, verbs, adjectives, pronouns, and adverbs are defined as content words. We calculated the *number of content words (CW)*, regardless of whether they appear in incomplete utterances or are used in grammatically incorrect forms—incomplete words it was not included in the content word count. *Content density* was measured as the ratio of CW to the TW. To analyze *the frequency of content words (CWF)*, we used the Academia Sinica Corpus of Contemporary Taiwan Mandarin (Lee, 2023), a database that contains Taiwanese Mandarin texts from 2015 to 2020, with a corpus exceeding 70 million words.

To assess syntactic complexity, we calculated several features, including *total utterances (U)*, *MLU*, *total sentences (S)*, *mean length of sentences (MLS)*, and features that indicate complex syntactic processing. Utterance is defined as the span of speech from the beginning of a speech to the subsequent pause or prosodic boundary. An utterance May be one word, a phrase, or a clause. Most Chinese words consist of two characters, but a word can be split into two utterances because of pauses. MLU was determined by dividing the total number of Chinese characters in an utterance by the total number of utterances. We counted complete sentences by identifying utterances that contained a subject or predicate structure: noun (or pronoun) + main verb or noun (or pronoun) + adjective phrase. To evaluate the production of complex sentences, we calculated the ratio of verbs and pronouns to the total number of CW, termed *verbal ratio (VR)* and *pronoun ratio (RP)*, respectively.

Mandarin Chinese uses morphosyntactic markers, such as *bèi* and *ba*, to indicate various grammatical relations and constructions, particularly passive constructions. For instance, the *bèi* construction typically arranges sentences in the following order: NP_1_ (object) + *bèi* + VP (verb phrase) [+ NP_2_ (agent)]. The use of *bèi* emphasizes the subject’s passivity in relation to the action described by the verb. Additionally, the *ba* construction presents a different syntactic structure: *ba* + NP (object) + VP. This structure effectively moves the object before the VP, emphasizing the action’s impact on the object and focusing on its outcome or result. These two passive construction markers in speech indicate the use of more complex sentence types and are therefore used in this study as indicators of syntactic complexity. To measure the use of passive construction, we calculated the ratio of passive markers to total utterances, termed the *passive construction ratio (PCR)*.

To calculate dysfluency, we assessed the ratios of fillers and long pauses to the TW in each speech sample, termed *filler ratios (FR)* and *long pause ratios* (LPR). Fillers such as “ah” and” um,” which are used to express hesitation or uncertainty, were meticulously recorded. Additionally, in Mandarin, words such as “this” and “that” can serve meaningful and filling purposes, and the transcribers relied on context to determine whether they functioned as fillers. Because of the meaningfulness of silent pauses, this study considered long pauses as those of 2 s or more between utterances (Lofgren and Hinzen, 2022; Pastoriza-Domínguez et al., 2022).

### 2.7 Data analysis

Statistical analysis was performed using R v4.3.2 (R Core Team, 2023) with the R packages *caret 6.0.94* (Kuhn, 2008) for machine learning modeling. Group differences were determined by comparing the NC and early-AD groups through linear regression models for each linguistic feature, adjusting for age and years of education as covariates.

#### 2.7.1 Classification model

The study aimed to distinguish the patients with early-AD from the NCs and identify the key linguistic features contributing to this classification and their association with cognitive functions. We employed three different predictor combinations to construct and compare predictive models: (1) Fifteen linguistic features; (2) two biomarkers (hippocampal volume and SUVR); and (3) fifteen linguistic features combined with the two biomarkers. In all three combinations, age and years of education were included as covariates. We employed three supervised learning algorithms: the support vector machine (SVM) model with a radial basis function kernel; the k-nearest neighbors (KNN) model, which classifies samples based on the majority class of their nearest neighbors; and the random forest (RF) model. The classification tasks were implemented using the *caret* package within the R programming environment. Feature selection was conducted using recursive feature elimination (RFE), an iterative method that removes the least informative features until the optimal subset is established. This process allowed us to identify the most relevant predictors for each model and predictor combination.

The dataset was randomly split into training (70%) and testing (30%) sets using stratified sampling. This process was repeated 10 times to create 10 different stratified train-test splits. For each split, the RFE process used 10-fold cross-validation repeated five times to select the optimal set of features. During this process, RFE evaluated feature subsets ranging in size from 1 to the total number of features in the training set. For each subset size, the performance was evaluated using the cross-validation procedure, allowing us to identify the optimal set of features based on the best accuracy across all evaluated subset sizes. After completion of the RFE process, we obtained a single optimal set of features, which was then used for training the final model on the entire training set and subsequently for making predictions on the test set. Models were then trained on each training set using only the features selected by RFE. The model training employed 5-fold cross-validation repeated 10 times for hyperparameter tuning using a grid search approach. For each model type—SVM with radial basis function kernel, KNN, and RF – we utilized the default hyperparameter tuning grids provided by the caret package. The SVM model’s cost (C) and sigma (*σ*) parameters, the KNN model’s number of neighbors (k), and the RF model’s number of variables randomly sampled as candidates at each split (mtry) were optimized. The receiver operating characteristic (ROC) curve was used as the performance metric for selecting the best hyperparameters. For each hyperparameter, 10 evenly spaced values within the default ranges were evaluated. Data preprocessing involved standardizing and centering to normalize the input features. Our predictive models’ efficacy was evaluated using various metrics, including area under the ROC curve (AUC), precision, recall, and F1 score. These metrics were assessed across the cross-validation folds applied to the test sets.

Additionally, the contribution of each variable to the models was estimated using the *varImp* function from the caret package, which calculates variable importance in a model-specific manner. Variable importance for the RF model was calculated using the mean decrease in Gini impurity, quantifying each variable’s contribution to node and leaf homogeneity. The SVM model with a radial basis function kernel assesses importance based on changes in the AUC when each predictor is removed. For the KNN model, which lacks an inherent importance measure, a model-independent permutation method is applied, measuring accuracy decrease upon random permutation of each predictor. All importance scores were scaled to a maximum value of 100 to facilitate comparison across models. The top five features were selected based on the mean importance value calculated across the models where the feature was selected.

#### 2.7.2 Regression models

We conducted linear regression analyses on 15 linguistic features to determine their effectiveness in predicting scores from MMSE, CDR-SB, and various neuropsychological tests (e.g., DS, WLM-I, WLM-II, CTT-I, CTT-II, VF, and BNT). Each test score was analyzed separately, with age and years of education included as covariates. We assessed the multicollinearity among the variables by using the variance inflation factor (VIF), excluding any variables with a VIF of greater than five to reduce the risks of instability and compromised interpretability. After examining nine regression models, we obtained similar results across them. This process led to the retention of 10—UW, TTR, CWF, MLU, MLS, VR, PR, PCR, FR, and LPR—of the 15 linguistic features. The residuals of the regression models were then examined to ensure that the models fulfilled all linearization assumptions. The regression tasks were implemented using the caret package, and feature selection was based on RFE Model optimization, guided by minimizing the root mean square error.

## 3 Results

Table 1 summarizes the demographic and cognitive characteristics of the NC and early-AD groups. On average, the ages of those in the early-AD group were greater than those in the NC group. Education levels differed significantly, with the NC and early-AD groups averaging 13.66 and 10.03 years of education, respectively. Gender distribution also varied, with females constituting 44 and 65% of the NC and early-AD groups, respectively. The average general cognitive performance assessed using MMSE scores was 28.94 and 21.19 for the NC and early-AD groups, respectively. CDR-SB scores differentiated the two groups: the NC group had a negligible average score of 0.02, and the early-AD group’s average score was 3.20, suggesting the early-AD group had more severe dementia symptoms than the NC group. Specifically, 75% of the early-AD group progressed and had elevated SUVR levels and significant hippocampal atrophy. Moreover, 58% of the early-AD group carried the ApoEε4 allele, significantly higher than the 13% in the NC group. These groups showed significant differences across all cognitive assessments, reflecting the effect of early-AD on different cognitive domains.

The recordings had an average length of 1.09 min per image. Table 2 compares the linguistic measures of the two groups. Syntactic complexity analysis showed that the MLU and MLS of the early-AD group were significantly shorter and used passive constructions less frequently than those of the NC group, indicating that the former used less complex speech patterns than the latter. Moreover, statistically significant differences between the two groups were observed in pronoun use. Disfluency measures showed a higher LPR in the early-AD group than in the NC group, indicating increased speech disfluency, and FR remained comparable in both groups.

### 3.1 Crucial features for classification

**Figure 2:**
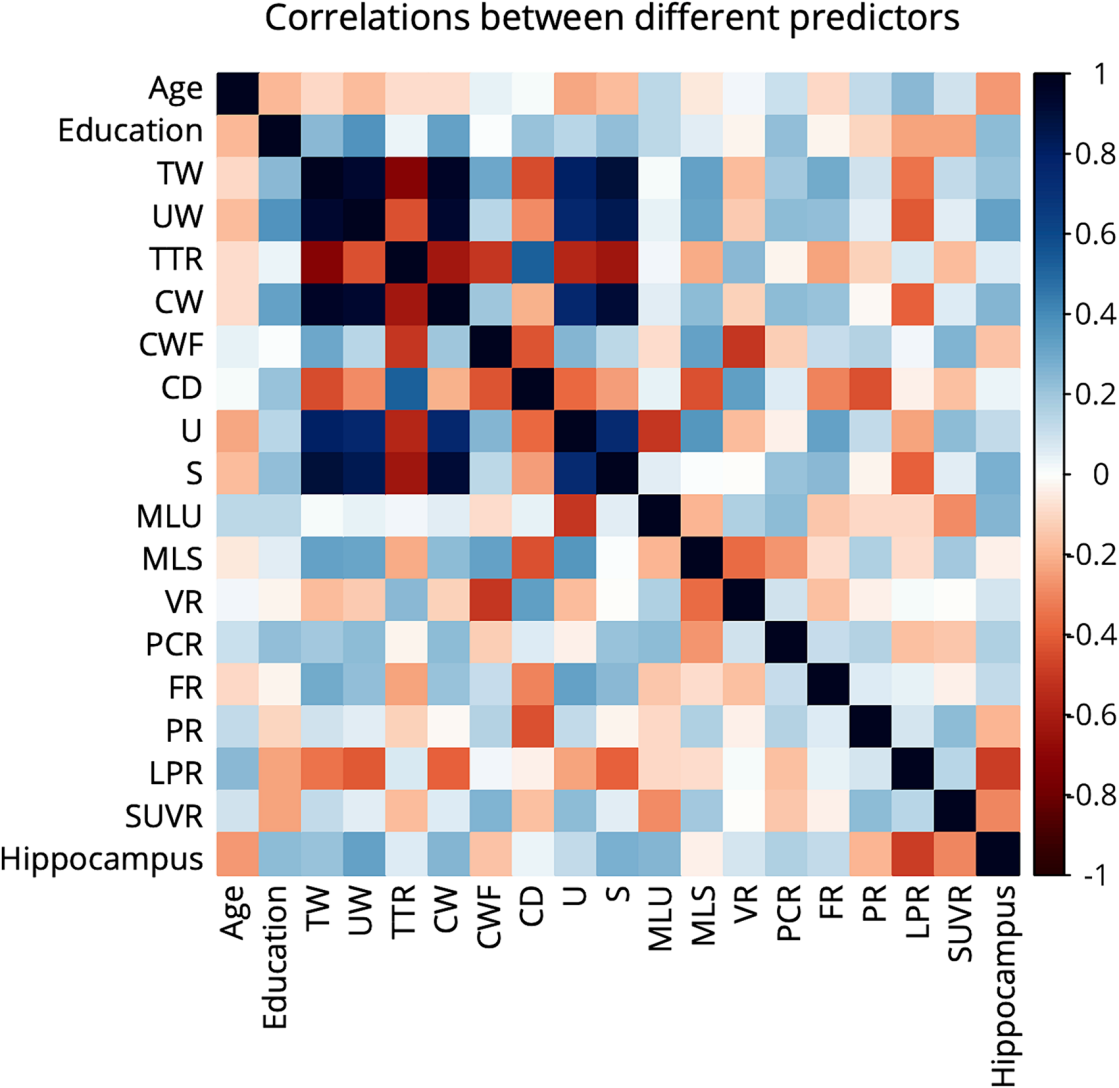
Correlation heatmap of linguistic features and biomarkers variables in early-AD detection. The heatmap displays Pearson correlation coefficients, with color intensity and hue indicating the strength and direction of correlations, respectively. Dark blue represents strong positive correlations (+1), while dark red indicates strong negative correlations (−1).

This study assessed the performance of the models in classifying early-AD by using linguistic features and biomarkers. Figure 2 presents a correlation heatmap illustrating the relationships among linguistic features, biomarkers, and demographic variables utilized in early-AD detection. The results show mostly weak correlations between biomarkers (Hippocampus volume and SUVR) and linguistic features, with a few notable exceptions. Hippocampus volume shows a moderate negative correlation with long pauses ratio (*r* = −0.489) and a moderate positive correlation with unique word count (*r* = 0.321).

**Figure 3:**
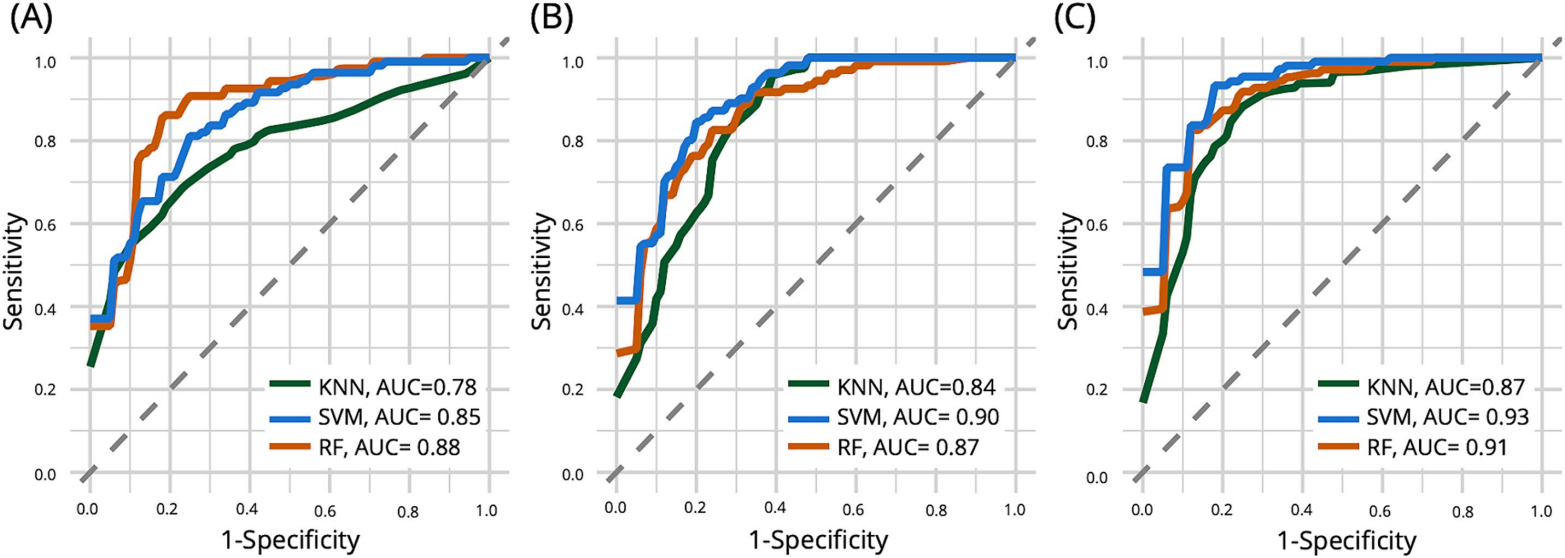
The curve of the test set for comparing the performance of three different classifier models: support vector machine (SVM), k-nearest neighbors (KNN), and random forest (RF). The left plot **(A)** shows that when only linguistic features are used, KNN shows the highest AUC. The middle plot **(B)** shows that KNN shows the highest AUC when using biomarkers alone. In the right plot **(C)**, including biomarker data alongside linguistic features substantially increased model performance for all three classifiers.

Table 3 presents the performance metrics of the early-AD classification for the SVM, KNN, and RF models. Figure 3 shows the ROC curve of the test set used to compare classifier performance. Table 4 presents the feature importance and selection frequency across different classifiers (SVM, KNN, and RF) and feature sets (Linguistic features, Biomarkers, and a combination of both). The additional classifier incorporating age and education demonstrated limited effectiveness, with AUC values nearing chance levels: SVM, 0.65; KNN, 0.65; and RF, 0.60. Education remained a notable factor in differentiating between the NC and early-AD groups across models. Although demographic factors provide some insights, linguistic features offer a more robust basis for differentiating between these groups.

**Table 3:** Performance metrics of machine learning models for early-AD classification using linguistic features and biomarker.

Features	Linguistic features			Biomarkers			Linguistic features & Biomarkers		
Classifiers	RF	SVM	KNN	RF	SVM	KNN	RF	SVM	KNN
AUC	0.88	0.85	0.78	0.87	0.90	0.84	0.91	0.93	0.87
Specificity	0.62	0.69	0.67	0.65	0.73	0.73	0.71	0.89	0.74
Precision	0.79	0.82	0.79	0.80	0.84	0.82	0.83	0.93	0.84
Recall (sensitivity)	0.89	0.84	0.78	0.85	0.86	0.76	0.90	0.84	0.81
F_1_ score	0.83	0.82	0.78	0.82	0.85	0.78	0.86	0.88	0.82

**Table 4:** Feature importance and selection frequency across different classifiers and feature sets.

	Linguistic features			Biomarkers			Linguistic features and biomarkers		
Classifiers	Feature	Importance	Frequency	Feature	Importance	Frequency	Feature	Importance	Frequency
SVM	LPR	93.42	100%	SUVR	100.00	100%	SUVR	98.62	100%
	MLU	75.60	100%	Hippocampus volume	81.56	100%	Hippocampus volume	84.72	100%
	Education	71.61	80%	Education	45.13	80%	LPR	74.01	100%
	PCR	52.48	75%	Age	19.61	11%	MLU	55.35	100%
	TW	37.03	50%				Education	54.76	100%
KNN	LPR	92.93	100%	SUVR	100.00	100%	SUVR	98.52	100%
	MLU	74.19	86%	Hippocampus volume	97.35	11%	Hippocampus volume	71.42	70%
	Education	46.22	60%	Education	79.20	100%	LPR	47.11	78%
	PR	32.24	100%				MLU	46.25	83%
	PCR	27.71	25%				PR	43.97	50%
RF	MLU	88.10	100%	SUVR	100.00	100%	SUVR	100.00	100%
	LPR	80.36	100%	Hippocampus volume	57.84	100%	MLU	62.50	100%
	PR	40.56	100%	Age	26.01	100%	LPR	53.33	100%
	TTR	31.77	100%				Hippocampus volume	52.21	100%
	Age	29.60	100%				PR	25.53	100%

Feature importance values represent the average over 10 random data splits. Selection frequency indicates the percentage of times each feature was selected and assigned non-zero importance across all model iterations and data splits.

When models were exclusively trained utilizing only linguistic features, the results indicated that the RF model exhibited the highest AUC at 0.88, followed by the SVM model with an AUC of 0.85 and the KNN model with an AUC of 0.78. All three models consistently identified MLU and LPR as crucial features in differentiating individuals with early-AD from those in the NC group, with high importance values across all models (MLU: 75.60–88.10; LPR: 80.36–93.42). The SVM model also highlighted Education and PCR as important features, with importance values of 71.61 and 52.48, respectively. Interestingly, the KNN and RF models identified PR as an additional important feature, albeit with lower importance values (KNN: 32.24, RF: 40.56).

Using biomarkers alone, the SVM model performed best with an AUC of 0.90, followed by the RF model (AUC = 0.87) and the KNN model (AUC = 0.84). All three models consistently identified SUVR as the most crucial biomarker, showing the highest importance (100.00) across all models. A combined approach involving biomarkers and linguistic features improved model performance. The SVM model achieved the highest AUC of 0.93, followed by the RF model (AUC = 0.91) and the KNN model (AUC = 0.87). In this combined approach, all three models consistently identified four crucial critical features: SUVR, Hippocampus volume, MLU, and LPR. SUVR consistently demonstrated the highest importance across all models (98.52–100.00), with Hippocampus volume (52.21–84.72) following as another significant predictor. Among the linguistic features, MLU (46.25–62.50) and LPR (47.11–74.01) emerged as the most critical, retaining their relevance across different classifiers.

### 3.2 Crucial features for predicting neuropsychological test scores

Linear regression was conducted to investigate the predictive capacity of linguistic features for cognitive impairment. The findings in Table 5 demonstrate a significant positive correlation between MMSE and linguistic features, including TTR and MLU. LPR and FR were positively associated with CDR-SB scores. TTR was associated with improved performance across various neuropsychological tests, particularly in memory (WLM-I, WLM-II, and DS) and language domains (BNT), and displaying negative correlations in the executive function domain (CTT1, CTT2, and VF). UW exhibited positive correlations with tests in both the memory (WLM-II and DS) and language domains (VF and BNT). Conversely, a higher CWF is associated with a lower performance on the BNT test. Regarding syntactic features, increased VR and decreased MLU were associated with poorer outcomes on the CTT-1 and CTT-2 tests. Additionally, higher PCR correlated with better performance on the WLM-I, DS, VF, and BNT tests. LPR displayed significant negative correlations with WLM-I and positive correlations with both CTT-1 and CTT-2.

**Table 5:** Linguistic features associated with cognitive functions.

	MMSE	CDR-SB	WLM-I	WLM-II	DS	CTT1	CTT2	VF	BNT
(Intercept)	24.29*** (0.49)	1.92*** (0.22)	8.82*** (0.43)	8.32*** (0.46)	9.04*** (0.42)	119.54*** (8.39)	206.77*** (10.22)	12.80*** (0.51)	21.54*** (0.36)
Age	−0.92 (0.59)	0.42 (0.26)	−1.13* (0.52)	−1.08 (0.55)			23.34* (11.43)	−1.72** (0.57)	−1.34** (0.39)
Education	0.96 (0.59)	0.21 (0.26)	0.60 (0.52)	0.89 (0.55)			19.18 (11.87)		
TTR	1.90* (0.79)	−0.64 (0.35)	1.48* (0.69)	1.74* (0.73)	2.04*** (0.50)	−40.74*** (9.05)	−55.89*** (15.79)	1.59* (0.70)	1.85*** (0.53)
UW	0.82 (0.74)	−0.37 (0.32)	1.13 (0.65)	1.46* (0.68)	1.31* (0.55)		−29.57 (15.30)	1.39* (0.68)	1.67** (0.49)
CWF	−0.66 (0.72)	0.38 (0.31)	−0.15 (0.63)	0.23 (0.66)			24.11 (14.54)		−1.07* (0.51)
VR	−0.54 (0.61)	0.53 (0.27)	−0.93 (0.54)	−0.60 (0.57)		17.66 (9.21)	28.44* (12.13)	−0.45 (0.57)	−0.83 (0.42)
PR	0.13 (0.53)	−0.21 (0.23)	−0.54 (0.47)	−0.59 (0.49)					−0.55 (0.37)
PCR	0.91 (0.61)	−0.26 (0.27)	0.97 (0.53)	1.14* (0.56)	1.01* (0.43)			1.46* (0.60)	1.35*** (0.38)
MLU	1.28* (0.63)	−0.44 (0.28)	0.74 (0.55)	1.12 (0.58)		−20.68* (8.59)	−29.09* (11.01)	0.92 (0.60)	
MLS	0.36 (0.59)	0.22 (0.26)	−0.06 (0.52)	−0.03 (0.55)					
LPR	−1.11 (0.59)	0.85** (0.26)	−1.17* (0.52)	−0.85 (0.55)	−0.92 (0.48)	41.78*** (8.49)	36.42** (12.13)	−0.81 (0.60)	−0.72 (0.42)
FR	−0.37 (0.60)	0.61* (0.27)	−0.73 (0.53)	−0.83 (0.56)				−1.18 (0.62)	
R^2^	0.49	0.49	0.49	0.51	0.34	0.40	0.51	0.42	0.61
AIC	477.78	345.98	456.83	465.47	443.82	924.69	960.00	479.98	423.13
BIC	511.12	379.33	490.18	498.82	458.11	938.98	983.82	503.80	446.95

All continuous predictors are mean-centered and scaled by 1 standard deviation. The outcome variable is in its original units. *** *p* < 0.001; ** *p* < 0.01; * *p* < 0.05. Bold values indicate statistically significant differences between the NC and Early-AD groups.

## 4 Discussion

Language deficits are crucial in identifying early-AD, often indicating progression to advanced stages. In this study, we examined the linguistic features of speech in patients with early-stage AD and healthy older adults. We aimed to distinguish between patients with early-AD and healthy older adults and elucidate how these features correlate with cognitive functions. Our analysis focused on identifying the critical linguistic features contributing to this classification and exploring their associations with cognitive functions. Consequently, our results provide insights into the potential diagnostic utility of speech analysis for early-AD detection.

We discovered that the early-AD group exhibited MLU and LPR, which were distinct from the NC group. Notably, the attributes differentiating the two groups showed reduced syntactic complexity in the patients with early-AD. The early-AD group was characterized by shorter utterances than those used in the NC group, and the former experienced challenges in constructing complex sentences, evidenced by their reduced use of passive constructions. Additionally, there was also a marked reduction in speech fluency for the early-AD group, characterized by long pauses that suggest word retrieval challenges. Furthermore, the early-AD group tended to use more high-frequency CW than the NC group did and frequently substituted concrete nouns with pronouns (“that” or “this”) to maintain fluent speech, pointing to a trend toward simplified language usage. These findings align with the observed decline in language specificity among patients with early-AD.

### 4.1 Crucial linguistic features for early detection of AD

In our study, we utilized SVM, KNN, and RF classifiers, leveraging MLU and LPR as core linguistic indicators. These features alone enabled strong predictive performance, with the RF classifier notably achieving an 88% detection accuracy on the test set. This outcome confirms that linguistic information extracted from speech serves effectively as a robust tool for AD prediction. Moreover, integrating these linguistic features with biomarkers enhanced the AUC across all classifiers tested. Notably, MLU ranked as the most influential predictor after the strong predictor SUVR, differentiating between the NC and early-AD groups and proving more decisive than hippocampus volume. This collaboration between linguistic features and biomarkers not only highlights the reliability of linguistic measures as crucial predictors but also emphasizes their role in boosting the accuracy of AD classification models.

An increase in the duration and frequency of pauses during speech is commonly observed with aging (Pastoriza-Domínguez et al., 2022). These pauses often serve as compensatory strategies to manage declining cognitive functions, with a significant increase in LPR indicating difficulties in lexical retrieval and memory degradation (Pastoriza-Domínguez et al., 2022; Pistono et al., 2016). In this study, long pauses are specifically defined as those exceeding 2 s during narrative speech, reflecting potential disruptions in speech generation. These abnormal pauses were rarely observed in the NC group, with an average LPR of 0.35%, and significantly more prevalent in the early-AD group (2%) than in the NC group. Similarly, Pistono et al. (2019) observed that participants with SD produced more pauses than healthy elders in the picture description task, correlating positively with semantic fluency and memory performance in the memory-related narrative tasks. In line with these findings, our study found that LPR correlated with cognitive decline in the early-AD group, particularly affecting short-term memory and executive functions.

Syntactic complexity is regarded as a higher-order aspect of cognitive function, with various syntactic units in speech reflecting distinct thoughts and capturing surface-level language characteristics (Lofgren and Hinzen, 2022). In our study, MLU served as a critical linguistic feature and an alternative measure of speech production complexity. We observed that MLU was notably shorter in the early-AD group than in the NC group, indicating a reduction in syntactic complexity in their speech. Correspondingly, Wang et al. (2022) found that patients with early-AD with preserved noun production ability often pause before nouns during discourse production. This study’s findings suggest that patients with early-AD May have had difficulty constructing complete sentences, highlighting their need to reform syntactic structures and editing breaks. As a result, patients with early-AD May produce simple or incomplete sentences and additional intra-sentence pauses.

Although some patients with mild AD can form simple sentences and produce a comparable number of utterances to healthy elderly and individuals with MCI, their speech shows significantly reduced syntactic complexity (Ivanova et al., 2023). In a related study, Sung et al. (2020) instructed NCs and patients with aMCI to identify target images corresponding to their heard sentences. The researchers manipulated syntactic complexity in sentences, and the participants listened to the active and passive sentences within the sentence-image paradigm. The results showed poorer accuracy in the aMCI group in processing passive sentences than in the normal aging group. The observed reduction in syntactic complexity might be associated with deficits in working memory and lexical retrieval (Nasiri et al., 2022).

Our study specifically measured the PCR as a Chinese language syntactic complexity marker. Our findings revealed that using passive constructions is a valuable indicator for classifying the early-AD group. These results prove that the PCR is an efficient marker of linguistic complexity, aiding in the early detection of cognitive-linguistic decline. In the context of Chinese language analysis, it’s worth noting that the calculation of PCR can be simplified by searching for specific markers indicating passive constructions. This approach is more efficient than parsing the entire SVO structure, especially in automated analysis systems.

Notably, the speech samples in our study were relatively short (i.e., approximately 110 words), which May have limited the ability to observe differences in lexical content. Longer speech samples than we used might explore more specific linguistic features (e.g., position or grammatical constituents) than we utilized in this study when assessing cognitive function. Therefore, further research could aim to expand these linguistic analyses with more extensive and diverse speech samples to validate and refine these diagnostic tools.

### 4.2 Linguistic features for predicting cognitive impairment

The findings from our regression models highlight the role of diverse linguistic features as critical indicators for assessing cognitive status in early-AD. Each feature correlates with specific cognitive functions, providing insights into AD’s progression and impact. Notably, TTR and MLU were associated with general cognitive functions, measured by the MMSE. These features also exhibited strong correlations with executive function assessments, suggesting their potential utility in detecting early cognitive impairments, such as MCI. Conversely, LPR was closely correlated with disease severity, measured by the CDR-SB. LPR is associated with both memory and executive function assessments, making it a valuable tool for monitoring disease progression. As AD advances, speech patterns show marked declines in fluency, evidenced by increased LPR. Our analysis also revealed that the PCR is linked to assessments in the memory (WLM-II, DS) and language domains (VF, BNT). As discussed in the literature, PCR is related to the ability to construct complex sentences, which is more cognitively demanding than active voice (Nasiri et al., 2022), and becomes increasingly challenging as AD progresses.

TTR and UW are two features that showed multiple significant relationships across different cognitive measures, most relating to single-word retrieval and working memory tasks, suggesting their importance as markers for word retrieval difficulties. Moreover, the increased CWF was primarily associated with poorer BNT test performance. An increase in CWF suggests a growing reliance on high-frequency words, potentially serving as a compensatory strategy for declining lexical access and retrieval abilities. These findings align with previous research (Forbes-McKay and Venneri, 2005; Kavé and Goral, 2016), which has consistently reported reduced lexical richness in individuals with AD.

In summary, our results largely parallel those from studies conducted in English and other Indo-European languages, pointing to universal aspects of cognitive decline in language production. However, it’s crucial to note that the specific manifestations of these effects differ between Mandarin and English due to their distinct syntactic structures and pronoun usage patterns. Mandarin, as a topic-prominent language, allows for subject omission in casual speech and employs less varied pronouns compared to English (Chen, 2004). These linguistic characteristics influence how complexity is expressed in connected speech, with potential implications for the development of language-specific cognitive assessment tools. While our study observed group differences in pronoun ratio, this feature did not emerge as a significant predictor in our models for early AD classification or cognitive impairment prediction. This finding May be influenced by our study’s sample size or the stage of cognitive decline in our participants. Further investigation, particularly through longitudinal studies tracking changes in pronoun usage over time, could provide valuable insights into the progression of language changes in AD and MCI.

This study has several limitations. First, we used a relatively small sample size, which May account for the variations in classification accuracy observed between groups. Thus, to build upon this work, further research should consider including a larger and more diverse cohort than we did. The educational level of participants in our early-AD group was 3 years lower than that of the control group. Despite adjusting such results via statistical methods, this discrepancy could still introduce bias into the speech performance of the early-AD group. Additionally, examining linguistic features across domains or those specific to Chinese speakers would enhance the assessment of cognitive decline domains or more accurately characterize various AD symptoms than we did. For example, incorporating tasks such as overlearned narrative recall, which involves a recollection of a familiar story by using complex sentence structures and multiple grammatical elements (Clarke et al., 2021), could be beneficial. Acknowledging the role of the distribution of pause duration in language production is also crucial (Lofgren and Hinzen, 2022; Pastoriza-Domínguez et al., 2022; Pistono et al., 2016). Finally, owing to the research scope, we did not investigate acoustic features; thus, they could be used in further research to improve the accuracy of early-AD detection further.

## 5 Conclusion

In conclusion, our research indicates the significance of MLU, LPR, and PCR as crucial for classification, while MLU, LPR, TTR, and PCR serve as key linguistic features for predicting cognitive impairments in Mandarin speakers with early AD. These findings support the potential of linguistic analysis as a tool for early AD detection and monitoring. However, we emphasize the importance of considering language-specific features, such as PCR in Mandarin, when developing diagnostic tools. Healthcare professionals can leverage the power of these linguistic features to employ efficient and reliable screening tools, considering the feasibility of integrating linguistic analysis tools in routine memory clinic assessments. Continued research should investigate the effectiveness and discriminatory power of MLU and LPR across various speech tasks and languages. Developing these two linguistic features for automatic measurement from connected speech would significantly enhance the early diagnosis of AD.

## Data Availability

The raw data supporting the conclusions of this article will be made available by the authors, without undue reservation.

## Footnotes

^1^ https://github.com/ckiplab
